# Bee Venom Alleviated Edema and Pain in Monosodium Urate Crystals-Induced Gouty Arthritis in Rat by Inhibiting Inflammation

**DOI:** 10.3390/toxins13090661

**Published:** 2021-09-16

**Authors:** Bonhyuk Goo, Jeeyoun Lee, Chansol Park, Taeyoung Yune, Yeoncheol Park

**Affiliations:** 1 Department of Acupuncture & Moxibustion, Kyung Hee University Hospital at Gangdong, Seoul 05278, Korea; goobh99@naver.com; 2 Age-Related and Brain Diseases Research Center, School of Medicine, Kyung Hee University, Seoul 02447, Korea; jeeyoun@khu.ac.kr (J.L.); chansol1028@khu.ac.kr (C.P.); tyune@khu.ac.kr (T.Y.); 3 Department of Biomedical Science, School of Medicine, Kyung Hee University, Seoul 02447, Korea; 4 Department of Biochemistry and Molecular Biology, School of Medicine, Kyung Hee University, Seoul 02447, Korea; 5 Department of Acupuncture & Moxibustion, College of Korean Medicine, Kyung Hee University, Seoul 02447, Korea

**Keywords:** bee venom, gouty arthritis, monosodium urate, colchicine, inflammation

## Abstract

Bee venom (BV) acupuncture has anti-inflammatory and analgesic effects; therefore, it was used as a traditional Korean medicine for various musculoskeletal disorders, especially arthritis. In this study, we investigated the effect of BV on monosodium urate (MSU) crystal-induced acute gouty rats. An intra-articular injection of MSU crystal suspension (1.25 mg/site) was administered to the tibiotarsal joint of the hind paw of Sprague Dawley rats to induce MSU crystal-induced gouty arthritis. Colchicine (30 mg/kg) was orally administered 1 h before MSU crystal injection as a positive control, and BV (0.5 mg/kg) was injected into the tibiotarsal joint immediately after MSU crystal injection. The ankle thickness, mechanical allodynia, and expression of proinflammatory cytokines (TNF-α, IL-1β, IL6, COX2 and iNOS) and chemokines (MIP-1α, MIP-1β, MCP-1, GRO-α, MIP-2α) were then evaluated. BV reduced the expression of proinflammatory cytokines and chemokines, which are important mediators of MSU crystal-induced inflammatory responses. This anti-inflammatory effect was also confirmed histologically to attenuate synovitis and neutrophil infiltration. We demonstrated that BV markedly ameliorated ankle edema and mechanical allodynia in gouty rats. These results suggest that BV acupuncture is a potential clinical therapy for acute gouty management.

## 1. Introduction

Gouty arthritis is an inflammatory rheumatoid joint disease caused by an inflammatory reaction due to the accumulation of monosodium urate (MSU) in the joints [1]. MSU crystals accumulated in the joints promote the release and synthesis of inflammatory mediators, thereby directly causing and amplifying the inflammatory response. In addition, cytokines, chemokines, proteases, and oxidants involved in the inflammatory response cause chronic gouty synovitis, cartilage loss, and bone erosion [2]. The major pathophysiological mechanisms of gouty arthritis are the recognition of MSU crystals by toll-like receptors, the uptake of MSU crystals by monocytes and synovial cells, the release of pro-inflammatory substances through NALP3 inflammasome activity, and inflammation progress due to neutrophil infiltration [3].

As major therapeutic agents used in clinical practice, colchicine (Col), nonsteroidal anti-inflammatory drugs (NSAIDs), and corticosteroids inhibit NALP3 inflammasome-induced caspase-1 activation and IL-1β release caused by MSU crystals in gouty arthritis, as well as inhibit chemotactic factors released from neutrophil lysosomes [4]. Although these treatments are used alone or in combination, there are side effects such as gastrointestinal disorders, exacerbation of heart failure (NSAIDs) [5], hypertension, restriction of use in diabetic patients (corticosteroids) [6], digestive side effects, and renal and nervous system toxicity (Col) [7,8]. Therefore, there is a great demand for a treatment that can quickly relieve pain and reduce inflammation while minimizing the side effects in the treatment of acute gouty arthritis.

Bee venom (BV) is a complex natural mixture with various pharmaceutical properties. It comprises a variety of peptides, enzymes, bioactive amines, and nonpeptide components. Melittin, Apamin, and phospholipase A2 (PLA2), the main components of BV, are the inflammatory peptides, and their effects are mediated by the inhibition of cyclooxygenase-2 (COX-2) and PLA2 [9]. For this reason, BV acupuncture was widely used for the treatment of clinical disorders such as neuropathic pain, intervertebral disc disease, spinal cord injury, musculoskeletal pain, and arthritis in traditional Korean medicine [10]. In particular, some cases of gouty arthritis were reported clinically [11,12], and only one experimental study reported that BV reduces levels of NF-κB nuclear translocation, leading to the suppression of proinflammatory cytokines such as TNF-α, IL-1β, and IL-6 in MSU-induced gouty mice [13].

However, the effective treatment doses and the mechanism by which BV modifies the clinical status of gouty arthritis are not well-known. In addition, previous studies using the intradermal paw injection model had limitations in reflecting gout attacks that cause pathological changes in the joints. For BV to be applied to the treatment of gouty arthritis, additional research is needed on the effect of bee venom on the inflammatory state of synovial tissue due to neutrophil infiltration. Therefore, this study was conducted to determine the effect of BV on various stages of the inflammatory response induced by MSU crystals using a model of intra-articular injection into the tibiotarsal joint with features that resemble gouty arthritis.

## 2. Results

### 2.1. BV Markedly Ameliorates Ankle Edema and Mechanical Allodynia in Gouty Rats

An MSU-induced animal model was established through tibiotarsal joint intra-articular injection therapy in Sprague Dawley (SD) rats. In a preliminary study, we measured paw edema and mechanical allodynia of the ipsilateral and the contralateral hind paw every 4 h for 24 h after MSU injection. It was confirmed visually that the hind paw injected with MSU was more swollen, and the ankle circumference was also increased. After 24 h, as the swelling subsided, the movement of the experimental animal became more active, and it was confirmed that mechanical allodynia was recovered in the same way as in the normal side (data not shown). This experiment was conducted in the same way as in the preliminary study, and edema and mechanical allodynia were evaluated for 24 h.

**Figure 1:**
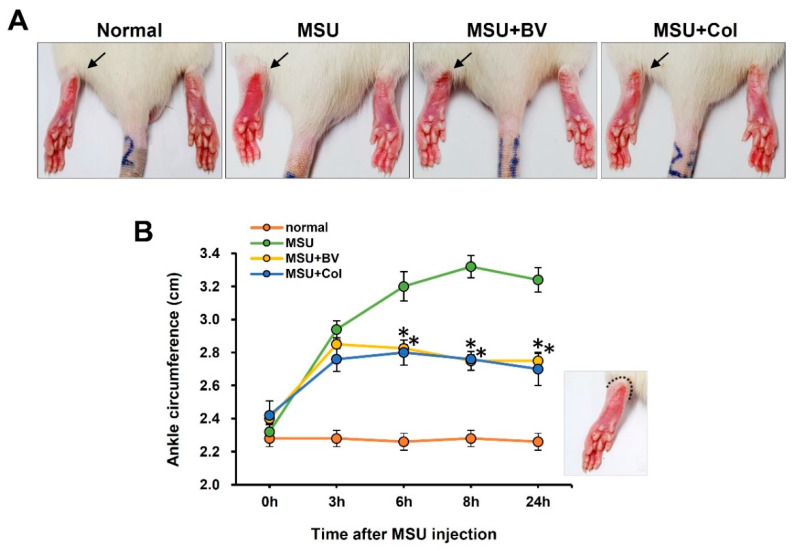
Effects of bee venom (BV) on monosodium urate (MSU) crystal-induced ankle edema in rats. Left hindpaw of rats were intra-articularly injected into the tibiotarsal joint with MSU crystal (1.25 mg/site). Immediately after MSU crystal injection, 50 μL BV (0.5 mg/kg) was injected in the same way. Colchicine (Col; 30 mg/kg) were orally administered 1 h before MSU crystal injection. (**A**) Representative photographs of hindpaw. Affected side (arrows). (**B**) Length of ankle circumference according to time after MSU crystal injection (*n* = 5/group). Data are presented as means ± SEM. * *p* < 0.05 vs. MSU group (repeated measures of ANOVA).

To evaluate the effect of BV in a gouty animal model, we checked the foot thickness of the rats. In the MSU injected group, there was a sharp increase for 3 h after injection, and then it became most severe after 8 h, after which it decreased gently for 24 h. In the case of the BV-treated and the Col-treated groups, the initial 3 h showed a similar pattern to the experimental group, and it decreased significantly until 24 h. These results suggest that BV mitigates MSU crystal-induced paw edema (Figure 1).

**Figure 2:**
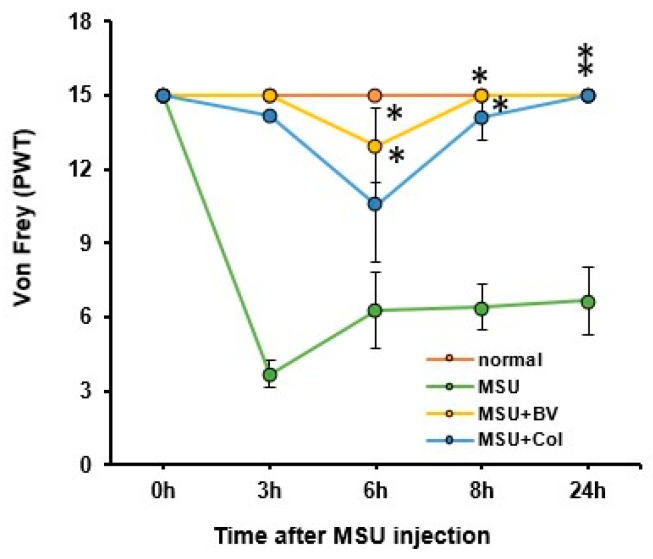
Effects of BV on MSU crystal-induced paw pain in rats. Comparison of paw withdrawal thresholds (PWTs) according to time after MSU crystal injection (*n* = 5/group). Data are presented as mean ± SEM. * *p* < 0.05 vs. MSU group (repeated measures of ANOVA).

We evaluated whether BV reduced the hind paw pain induced by MSU crystals. Paw withdrawal threshold (PWT) was measured for 24 h after MSU injection using the von Frey test. In the MSU-only treated group, it decreased by about 80% compared to that of the normal group after 3 h, and it increased slightly for 24 h (60% of normal value). In the BV-and Col-treated groups, there was no rapid decrease in PWT for the first 3 h, and after 6 h, it decreased by approximately 13% (BV-treated) and 23% (Col-treated), respectively. After 24 h, both groups returned to normal levels. A significant difference was observed between the two groups compared to the MSU injected group (Figure 2).

### 2.2. BV Attenuate Infiltration of Neutrophil in Gouty Rats

**Figure 3:**
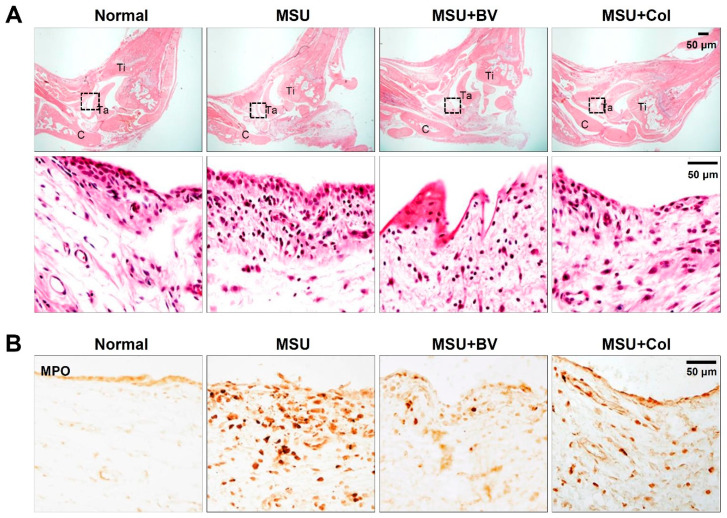
Effects of BV on infiltration of neutrophil in gouty rat. (**A**) Representative hematoxylin and eosin (H&E) stained sections of ankle joint from normal, MSU only, MSU with BV, and MSU with Col treated group. Purple dots represent neutrophils infiltration. Bottom panels are a high magnification of boxed area in the upper panels, which is synovial tissue of ankle joint. Scale bars, 50 μm. (**B**) Immunohistochemistry for myeloperoxidase (MPO) in ankle joint from normal, MSU only, MSU with BV, and MSU with Col treated group. MPO-positive neutrophils were observed in MSU injected rat ankle joint. Note that both BV and Col treatment decreased neutrophil recruitment. Scale bars, 50 μm.

To investigate the effect of BV on reducing synovitis and inhibiting neutrophil infiltration in MSU crystal-induced gouty rats, hematoxylin and eosin (H&E) and immunohistochemistry were performed. Results of H&E staining of synovial tissue in the ankle joint showed a slight synovitis pattern (slight enlargement of the synovial lining cell layer, increase in stroma cell density, and inflammatory infiltration) in the MSU injected group. However, the condition of synovitis was attenuated in the BV- and Col-treated groups compared to that of the MSU injected group (Figure 3A). In addition, myeloperoxidase (MPO) positive neutrophils (dark brown) were remarkably increased in the MSU injected group at 24 h after MSU crystals injection. However, BV or Col treatment markedly inhibited the infiltration of neutrophils compared with the MSU injected group (Figure 3B). These results suggest that BV suppresses neutrophil infiltration following MSU injection and reduces the progression of synovitis.

### 2.3. BV Reduces the Expression of Proinflammatory Cytokines and Chemokines in Gouty Rats

**Figure 4:**
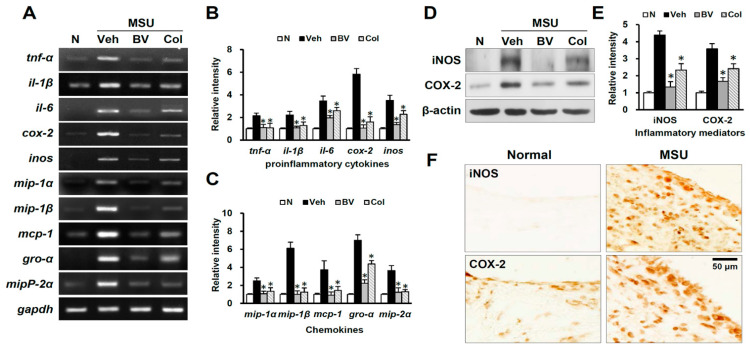
Effects of BV on proinflammatory cytokines and chemokines in gouty rat. (**A**) Reverse transcriptase PCR of proinflammatory cytokines (TNF-α, IL-1β, IL6, COX2 and iNOS) and chemokines (MIP-1α, MIP-1β, MCP-1, GRO-α, MIP-2α) (at 24 h) in normal, vehicle-treated, BV-treated, and Col-treated rats (n = 3/group). (**B**,**C**) Quantitative analysis of reverse transcriptase PCR. (**D**) Western blots of iNOS and COX2 at 24 h in normal, vehicle-treated, BV-treated, and Col-treated rats (n = 3/group). (**E**) Quantitative analyses of western blots. (**F**) Immunohistochemistry for iNOS and COX2 in synovium tissue of ankle. Scale bar, 50 μm. Data are presented as means ± SD. * *p* < 0.05 versus vehicle (one way ANOVA).

Blood cell infiltration following MSU injection initiates inflammatory responses by producing inflammatory mediators such as tumor necrosis factor (TNF)-α, interleukin (IL)-1β, IL-6, COX-2, and inducible nitric oxide synthase (iNOS). Therefore, we examined the effect of BV on blood cell infiltration and expression of inflammatory mediators by reverse transcriptase-polymerase chain reaction (PCR), western blotting, and immunohistochemistry at 24 h after MSU injection, and found that the messenger RNAs levels of TNF-α, IL-1β, IL6, COX-2, and iNOS (at 24 h) were significantly upregulated after MSU injection, but BV and Col significantly reduced their levels as compared with that of MSU injected rats (*n* = 3) (Figure 4A,B). The expression of chemokines such as GRO-α (growth-regulated oncogene-α; CXCL1), MCP1 (monocyte chemoattractant protein-1; CCL2), and MIP-1α (macrophage inflammatory protein-1α; CCL3) is known to induce the infiltration of blood cells, thereby facilitating inflammatory responses [14]. Thus, we examined the effect of BV on the expression of chemokines such as MIP-1α, MIP-1β (CCL4), MCP-1, GRO-α, and MIP-2α (CXCL2) by reverse transcriptase-PCR (*n* = 3). As shown in Figure 4A,C, the MIP-1α, MIP-1β, MCP-1, GRO-α, and MIP-2α messenger RNAs increased at 24 h after MSU injection. Furthermore, messenger RNA expression of MIP-1α, MIP-1β, MCP-1, GRO-α, and MIP-2α at 24 h after MSU injection was significantly inhibited by BV and Col treatment. By western blotting, the protein levels of inducible iNOS and COX-2 at 24 h after MSU injection were significantly decreased by BV and Col as compared with that of MSU injected group (*n* = 3, P) (Figure 4D,E). Immunohistochemistry results also showed that after MSU injection, the number of iNOS- and COX-2-immunoreactive cells was increased in the synovial tissue of the ankle joint (Figure 4F). These results suggest that BV may inhibit of inflammatory responses in MSU crystal-induced gouty rats, and might reduce the number of infiltrating blood cells by attenuating the expression of chemokines.

## 3. Discussion

In this study, we demonstrated that BV, an anti-inflammatory natural toxin, relieves edema and mechanical allodynia by preventing neutrophil infiltration and inhibiting pro-inflammatory cytokines and chemokines after MSU crystals induce gouty arthritis. Furthermore, our results showed that BV reduced the number of infiltrating blood cells such as neutrophils after MSU injection by suppressing the expression of chemokines such as MIP-1α, MIP-1β, MCP-1, GRO-α, and MIP-2α. These results suggest that BV reduces the inflammatory response following MSU injection.

A gouty model was successfully created through intra-articular injection into the tibiotarsal joint, and the evaluation timeline of this study was derived from our preliminary study on changes in edema and mechanical allodynia for 24 h. According to previous studies, the gouty model showed various patterns of swelling and pain according to the number and dose of MSU injections [15,16,17,18]. In our study, wherein 1.25 mg MSU dose was injected, the edema and pain patterns were similar to those of a previous study [18].

After MSU crystals are recognized by toll-like receptor 2/4 in the early stages of gouty arthritis [2], uptake of MSU crystals by macrophages, mast cells, and synovial lining cells activates the NALP3 inflammasome [19]. As a result, various cytokines and chemokines are released, and in particular [20], IL-1β and TNF-α promote the recruitment of neutrophils, resulting in the inflammation of gouty arthritis through the release of a large amount of proinflammatory substances such as IL-1β, TNF-α, and IL-6 [21]. Our results showed that the expression of IL-1β, TNF-α, and IL-6 was increased in the gouty rats, and the inhibitory effect of BV was similar to or better than that of Col, a treatment drug for acute gouty arthritis.

Inflammatory cytokines affect COX-2 expression, resulting in increased Prostaglandin E2 (PGE2) production, which is related to inflammation and osteoclastic activity [22]. iNOS, a form of nitric oxide synthase, is an enzyme that controls inflammation. Increased iNOS activity upregulates nitric oxide (NO) production [23]. Since excessive NO and PGE2 production causes inflammatory reactions and tissue damage, inhibition of COX-2 and iNOS, which triggers PGE2 and NO production, is one of the main targets for the inhibition of inflammation [24]. In our study, immunohistochemical analysis confirmed that iNOS and COX-2 were upregulated after MSU crystal injection, and showed that BV suppressed their expression.

Acute inflammation, such as gouty arthritis, is a complex process characterized by the coordinated migration of effector cells and immune cells to the site of inflammation. Such coordinated movement of cells requires induced expression of inflammatory chemokines. In particular, neutrophil infiltration at the site of injury plays an important role in the inflammatory response [25]. Adhesion molecules, whose expression is increased on the surface of the capillary blood vessels due to an inflammatory response, react with integrins on the surface of neutrophils, causing the neutrophils to attach to the walls of capillaries [26]. Neutrophils secretes MIP-1β after adhering to laminin, and MIP-1β induces the chemotaxis of dendritic cells [27]. Additionally, other products of inflammation cause chemotaxis in neutrophils, allowing them to infiltrate the damaged tissue. MCP-1 and MIP-1α mediate neutrophil recruitment, an essential step in the inflammatory response, via induction of protein synthesis and generation of lipid mediators [14], and GRO-α and MIP-2α induce neutrophil infiltration via macrophage toll-like receptor signaling [28]. Our study demonstrated that BV effectively inhibited the expression of chemokines such as MIP-1α, MIP-1β, MCP-1, GRO-α, and MIP-2α, which induce various blood infiltrations, including neutrophils, in the inflammatory response.

In gouty arthritis, neutrophils undergo a process of neutrophil extracellular traps (NETs) consisting of DNA, histones, and neutrophil granules (including neutrophil elastase and MPO) to respond to stimulation by MSU crystals [29]. NETs induced by MSU crystals can induce proinflammatory responses. Thus, neutrophils and/or NETs play an important role in the initiation and resolution of acute inflammatory diseases, such as gouty arthritis [30]. Intense infiltration of neutrophils into the synovial membrane and fluids is a hallmark of acute gout. MSU crystal-induced synovitis can be inhibited by depletion of neutrophil [31]. We showed that BV and Col decreased the increase in MPO-positive cells and decreased MSU-induced synovitis in the ankle joint tissue of gouty rats.

Severe joint pain and edema are hallmarks of the acute gouty arthritis. The primary goal in the treatment of acute gouty arthritis is rapid pain reduction and improvement of edema [21]. During acute gouty attacks, vasodilation, erythema, and pain are promoted by various mediators released by MSU crystals and neutrophil interactions. This pain may be due to several factors including local production of prostaglandins and bradykinin, and sensitization of nociceptors [32]. In our study, we showed that ankle edema in MSU crystal-induced gouty rats was reduced by BV, similar to Col, and that BV minimized the severity of mechanical allodynia and resulted in faster recovery.

In conclusion, BV induced marked improvement in mechanical allodynia and edema induced by MSU crystals to a similar degree or more effectively than Col, a representative treatment for acute gout attacks. The alleviating effect of BV appears to be achieved by suppressing various steps required for the activation of inflammation induced by MSU crystals, including MSU recognition, the release of proinflammatory substances according to the activation of the inflammasome, and the recruitment of neutrophils through blood infiltration-induced mediators. This study has limitations as it is a study that confirmed the effect of BV on the most acute gout attacks. For BV to be used for the treatment of gouty arthritis, it is necessary to study the effects of BV in subacute and chronic gouty models, such as a gouty model through low-dose and repeated MSU crystal administration.

## 4. Materials and Methods

Purified BV was purchased from Chungjin Biotech (Ahnsan, Korea). Table 1 shows the results of the analysis of BV components. MSU crystals (25 mg, Cat. No. tltl-msu-25) were purchased from Invivogen (San Diego, CA, USA), and 0.6 mg Col tablets (Korea United Pharm Inc., Seoul, Korea) were prescribed for the research in Kyung Hee University Hospital at Gangdong (Gangdong-gu, Seoul, Korea) required doses and used in the experiment.

### 4.1. Animals

Adult male SD rats (7 weeks old, 200–240 g; Samtako, Osan, Korea) were individually housed with water and food available ad libitum. The room maintained a 12-h light/dark cycle and remained at a constant temperature of 21–24 °C. All experiments were conducted according to the “Guiding Principles for the Care and Use of Laboratory Animals”, and all procedures were approved by the Animal Care and Use Committee of Kyung Hee University Hospital at Gangdong (approval number: KHNMC AP 2019–011).

### 4.2. MSU Crystals-induced Acute Gouty Arthritis Animal Model

The gouty model was induced by intra-articular injections of MSU based on a previously described method with minor modifications. [18]. Rats were anesthetized with chloral hydrate (500 mg/kg), and 1.25 mg MSU (in 100 μL phosphate-buffered saline) was injected into the left tibiotarsal joint (ankle). The injection point corresponds to the Jiexi (ST41) in traditional Korean medicine, and the injection technique used in the experiment was performed in the same way as the intra-articular acupuncture technique of the Jiexi (ST41). Observations were made hourly for 24 h to evaluate edema and mechanical allodynia. To confirm the efficacy of BV, gouty arthritis was induced in the same way as in the preliminary experiment, and then the experiment was conducted by dividing it into four groups, which consisted of five animals each, as follows: control group, MSU-injected group with MSU crystal injection only, BV-treated group with MSU crystal injection, and Col-treated group with MSU crystal injection. Immediately after MSU crystal injection, 50 μL BV (0.5 mg/kg) was administered via tibiotarsal intra-articular injection [13]. Col (30 mg/kg) was orally administered 1 h before the MSU crystal injection as a positive control [18]. Phosphate-buffered saline was administered as a vehicle control in the same way. In our previous experiment, in which BV alone was injected intra-articularly, pain and edema increased temporarily (after 3 h), but returned to normal within 6 h. No significant systemic side effects resulting from BV treatment, such as changes in body weight or increase in mortality, were observed during our experiments.

### 4.3. Assessment of Ankle Edema and Mechanical Allodynia

To determine ankle edema, the diameter of left ankle was measured as shown in the Figure 1B. Mechanical allodynia was assessed using von Frey filaments (Stoelting, Kiel, WI, USA). The left hind paw was pressed with a series of von Frey filaments (0.4, 0.6, 1.0, 2.0, 4.0, 6.0, 8.0, and 15.0 g). The 50% PWT was determined by using the up-down method as previously described [33]. Stimuli were applied to the hindpaw for 3–4 sec while the filament was bent, and were presented at several second intervals. A rapid hind paw withdrawal in response to the application of von Frey filament was considered a positive response. All testing was performed by trained investigators who were blind as to the experimental conditions.

### 4.4. Tissue Preparation and Histological Analysis

For histological analyses, the rats were sacrificed 24 h after MSU injection. The ankle region was isolated and fixed in 4% paraformaldehyde for 2 days, decalcified in Calci-Clear Rapid solution (National Diagnostics, Atlanta, GA, USA) for 10 days, and then embedded in paraffin. The samples were sectioned at 5 μm for staining with H&E and immunohistochemical staining. The slides were stained with hematoxylin for 5 min and eosin for 3 min at room temperature and observed under a light microscope (magnification, x40 and x200), and three fields of view were examined per section. For molecular analysis, isolated ankle regions were frozen at -80 °C.

### 4.5. RNA Isolation and Reverse Transcription-PCR

Twenty four hours after MSU injection, total RNA was extracted from the ankle syno-vial tissue (300 mg) of rats treated with vehicle (saline), BV or Col using TRIZOL Reagent (Invitrogen, Carlsbad, CA, USA). RNA was quantified using the NanoDrop ND-2000 (Thermo Fisher Scientific, Carlsbad, CA, USA), and reverse transcripts were synthesized using the PrimeScript 1st strand cDNA Synthesis Kit (Takara Bio, Shiga, Japan) from 1 µg of total RNA. A 20 μL PCR reaction contained 1 μL first strand cDNA, 0.5 U taq polymerase (Takara, Kyoto, Japan), 20 mM Tris-HCl, pH 7.9, 100 mM KCl, 1.5 mM MgCl2, 250 μM dNTP, and 10 pmole of each specific primer. The primers used for TNF-α, IL-1β, IL-6, COX-2, iNOS, MIP-1α, MIP-1β, MCP-1, GRO-α, MIP-2α and glyceraldehyde 3-phosphate dehydrogenase (GAPDH) were synthesized by the Genotech (Daejeon, Korea), and the sequences of the primers are presented in Table 2. After amplification, PCR products were subjected to a 1.5 or 2% agarose gel electrophoresis and visualized by ethidium bromide staining. The relative density of bands (relative to normal value) was analyzed using the AlphaImager software (Alpha Innotech Corp. San Jose, CA, USA). Experiments were repeated three times, and the values obtained for the relative intensity were subjected to statistical analysis. The gels shown in figures are representative of results from three separate experiments.

### 4.6. Western Blot

Total protein was prepared as previously described [34]. Tissue homogenates were incubated for 20 min at 4 °C, and centrifuged at 25,000× *g* for 30 min at 4 °C. The protein concentration was determined using the bicinchoninic acid protein assay kit (Pierce, Rockford, IL, USA). Protein samples (30 μg) were separated on sodium dodecyl sulphate–polyacrylamide gel electrophoresis and transferred to nitrocellulose membrane (Millipore, Burlington, MA, USA). The membranes were blocked in 5% nonfat skimmed milk or 5% bovine serum albumin in Tris-buffered saline solution with Tween (TBST) for 1 h at room temperature followed by incubation with antibodies against iNOS (1:10,000, Transduction Laboratory, Lexington, KY, USA), COX-2 (1:1000, Cayman Chemicals, Ann Arbor, MI, USA), and β-actin (1:30,000; Sigma, St. Louis, MO, USA).

Primary antibodies were detected using horseradish peroxidase-conjugated secondary antibodies (Jackson ImmunoResearch, West Grove, PA, USA). Immunoreactive bands were visualized by chemiluminescence using SuperSignalTM (Thermo Scientific, Waltham, MA, USA). β-actin was used as an internal control. Experiments were repeated three times, and the densitometric values of the bands on western blots obtained using AlphaImager software (Alpha Innotech Corp., San Leandro, CA, USA) were subjected to statistical analysis. The background of the films was subtracted from the optical density measurements.

### 4.7. Immunohistochemistry

Frozen sections were processed for immunohistochemistry with antibodies against MPO (1:100; Dako, Carpinteria, CA, USA), iNOS (1:500, Transduction Laboratory) and COX-2 (1:200, Cayman Chemicals, Ann Arbor, MI, USA), as previously described [34]. The sections were incubated with primary antibodies, followed by biotin-conjugated secondary antibodies (Dako). The ABC method was used to detect labeled cells using a Vectastain kit (Vector Labs, Burlingame, CA, USA). Diaminobenzidine served as the substrate for peroxidase. Immunostaining control studies were performed by omission of the primary antibodies, by replacement primary antibodies with nonimmune, control antibody, and by preabsorption with an excess (10 µg/mL) of the respective antigens.

### 4.8. Statistical Analysis

Data are presented as mean ± SD or mean ± SEM. Multiple comparisons between normal, MSU injected, BV-treated, and Col-treated groups were performed using one-way analysis of variance (ANOVA). The length of ankle circumference and von Frey tests were analyzed using repeated measures of ANOVA (time vs. treatment). Tukey’s multiple comparison test was used for posthoc analysis. Statistical significance was set at *p* < 0.05. All statistical analyses were performed using SPSS (version 15.0, SPSS Science, Chicago, IL, USA).

## Figures and Tables

**Table 1:** Analysis on components of BV.

Test	Specification	Method	Result
**Physical assay**			
Appearance	Clear powder	Visual	Pass
Color	Beige	Visual	Pass
Solubility	Soluble in water		Pass
Density	1.1–1.4		1.13
pH value	5.1–5.5	Orion pH meter	5.25
**Chemical Assay**			
Melittin	45–65%	HPLC	65.00%
Apamin	2.0–3.0%	HPLC	2.74%
PLA2	11.0–13.0%	HPLC	12.27%
Heavy metals	≤1.0 ppm	AOAC	Conform
Total ash	≤4.0%	AOAC	Conform
**Microbial Assay**			
Aerobic plate count	Negative	Petrifilm 3 M	Conform
Mold & yeast	Negative	Petrifilm 3 M	Conform
*S. aureus*	Negative	Petrifilm 3 M	Conform
**Histamine content**		Histamine Enzymatic assay kit	1.75 ppm,
**Endotoxin test**	≤0.25 EU/mL	EndoSafe^®^-PTS	0.09 EU/mL

Analysis results were provided by Chongjin Biotech. (www.biovenom.com, accessed on 3 August 2021).

**Table 2:** Nucleotide sequences of primers and conditions used for RT-PCR.

Target	Primer	Sequence	Annealing Temperature (℃)	Cycles
TNF-α	Forward	5′-CCC AGA CCC TCA CAC TCA GAT-3′	58	30
	Reverse	5′-TTG TCC CTT GAA GAG AAC CTG-3′		
IL-1β	Forward	5′-GCA GCT ACC TAT GTC TTG CCC GTG-3′	52	35
	Reverse	5′-GTC GTT GCT TGT CTC TCC TTG TA-3′		
COX-2	Forward	5′-CCA TGT CAA AAC CGT GGT GAA TG-3′	58	35
	Reverse	5′-ATG GGA GTT GGG CAG TCA TCA G-3′		
iNOS	Forward	5′-CTC CAT GAC TCT CAG CAC AGA G-3′	59	30
	Reverse	5′-GCA CCG AAG ATA TCC TCA TGA T-3′		
MIP-1α	Forward	5′-ACT GCC TGC TGC TTC TCC TAC A-3′	62	35
	Reverse	5′-AGG AAA ATG ACA CCT GGC TGG-3′		
MIP-1β	Forward	5′-TCC CAC TTC CTG CTG TTT CTC T-3′	60	35
	Reverse	5′-GAA TAC CAC AGC TGG CTT GGA-3′		
MCP-1	Forward	5′-TCA GCC AGA TGC AGT TAA CG-3′	55	30
	Reverse	5′-GAT CCT CTT GTA GCT CTC CAG C-3′		
GRO-α	Forward	5′-CCG AAG TCA TAG CCA CAC TCA A-3′	65	35
	Reverse	5′-GCA GTC GTC TCT TTC TCC GTT AC-3′		
MIP-2α	Forward	5′-AGA CAG AAG TCA TAG CCA CTC TCA AG-3′	32	35
	Reverse	5′-CCT CCT TTC CAG GTC AGT TAG C-3′		
GAPDH	Forward	5′-AAC TTT GGC ATT GTG GAA GG-3′	50	25
	Reverse	5′-GGA GAC AAC CAG GTC CTC AG’		

## Data Availability

The data presented in this study are available on request from the corresponding author.
